# Unlocking the Potential of Deep Eutectic Solvents for C–H Activation and Cross-Coupling Reactions: A Review

**DOI:** 10.3390/molecules28124651

**Published:** 2023-06-08

**Authors:** Yassine El Baraka, Ghanem Hamdoun, Nabil El Brahmi, Saïd El Kazzouli

**Affiliations:** Euromed Research Center, Euromed Faculty of Pharmacy, School of Engineering in Biomedical and Biotechnology, Euromed University of Fes (UEMF), Meknes Road, Fez 30000, Morocco; y.elbaraka@biomedtech.ueuromed.org (Y.E.B.); g.hamdoun@ueuromed.org (G.H.); n.elbrahmi@ueuromed.org (N.E.B.)

**Keywords:** deep eutectic solvents, green chemistry, C–H activation, cross-coupling, sustainability, recyclability

## Abstract

Green chemistry principles have underpinned the development of deep eutectic solvents (DESs). In this brief overview, we discuss the potential of DESs as a greener alternative to volatile organic solvents for cross-coupling and C–H activation reactions in organic chemistry. DESs offer numerous benefits, such as easy preparation, low toxicity, high biodegradability, and the potential to replace volatile organic compounds. The ability of DESs to recover the catalyst-solvent system enhances their sustainability. This review highlights recent advances and challenges in utilizing DESs as a reaction media, as well as the impact of physicochemical properties on the reaction process. Several types of reactions are studied to highlight their effectiveness at promoting C–C bond formation. Aside from demonstrating the success of DESs in this context, this review also discusses the limitations and future prospects of DESs in organic chemistry.

## 1. Introduction

The use of solvents is a major concern in applying sustainable principles in the chemical industry. Solvents are widely used for various applications, such as coatings, paints, product synthesis, adhesives, equipment cleaning, and reaction media [1,2,3]. The selection of an appropriate solvent is crucial for a chemical process [4,5]. Traditionally, volatile organic derivatives have been used as the solvents, which are non-renewable, highly toxic, non-degradable, and accumulate in the atmosphere due to their low-boiling points, which contribute to a high-carbon footprint [6,7,8,9,10,11]. Recent reports have highlighted that solvents constitute 80–90% of the non-aqueous content in materials used for producing active pharmaceutical substances and fine chemicals [12]. Therefore, it is vital to discover substitute solvents to enhance the sustainability of these industries.

In this context, DESs (Deep Eutectic Solvents) have been proposed as alternative solvents in organic chemistry. They have emerged as a potential class of solvents for various organic chemistry applications. DESs are characterized as systems resulting from a eutectic mixture of two or more components, typically Lewis or Brønsted acids and bases, that encompass diverse anionic and/or cationic species [13]. The formation of DESs involves intermolecular interactions between their constituents, facilitated by various types of bonds, including but not limited to hydrogen bonding. These interactions result in a reduction of lattice energy, thereby contributing to the lowered melting point of DESs, as compared to their individual components [14]. Eutectic mixtures can be prepared by combining the constituent components in the correct proportions and then heating the mixture until it melts [15]. This method is highly efficient in terms of atom economy, with no by-products generated. Compared to similar ionic liquids, DESs offer advantages, such as easy preparation and high-atom economy. DESs also have other beneficial properties, such as low-boiling points, low-material costs, being sourced from renewable sources, having low toxicity, high biodegradability, and the potential to replace volatile organic compounds (VOCs) in organic reactions [16]. Therefore, DESs hold significant promise as a potential alternative to VOCs in organic reactions. They can be classified into five main types based on their composition:

▪ Type I: composed of a metal chloride and a quaternary ammonium salt.
▪ Type II: similar to type I, but with hydrated metal halides instead of non-hydrated ones.
▪ Type III: composed of a hydrogen bond donor (HBD), such as alcohols, amino acids, or amides, and a quaternary ammonium salt.
▪ Type IV: composed of a transition-metal salt and HBDs.
▪ Type V: composed solely of non-ionic components.

C–H activation is a process that enables the direct functionalization of carbon–hydrogen bonds, without requiring pre-functionalization. On the other hand, cross-coupling reactions can join a wide range of organic molecules, including aryl halides, vinyl halides, aryl triflates, boronic acids or boronates, alkynes, and alkyl halides, to form a new molecule with a carbon–carbon bond. There are numerous publications, such as articles and reviews, that discuss C–H activation and cross-coupling reactions [17,18,19,20,21,22,23,24,25,26,27,28,29,30]. These reactions have been extensively studied and widely used in organic synthesis, making them a popular research topic in chemistry. While OVSs have been widely used as reaction media for this type of reaction, there has been growing interest in exploring the potential of DESs as a greener alternative. In catalytic transformations, it is crucial to ensure that the catalyst used is compatible with the DESs employed to produce the desired products with selectivity and efficiency. Sometimes, transition-metal salts can serve as the catalysts due to the high polarity of DESs.

One key property of DESs is their ability to activate electron rich substrates, including carbohydrates, amino acids, and enzymes. DESs often have an electron deficiency, which can be compensated for by the electron rich substrate. When the DES is brought into close proximity with the substrate, electrons can be transferred from the substrate to the DES. As a result, the substrate is activated through a process known as electron transfer activation. By activating substrates, DESs have improved catalytic activity, making them attractive for use in a variety of chemical reactions and processes [31]. One significant advantage of DESs over organic volatile compounds is their ability to recover the catalyst-solvent system, allowing for recyclability and increasing the sustainability of the process. This review highlights recyclability by quantifying reaction cycles for each example. DESs surpass organic volatile compounds in dissolving reagents and substrates, making them excellent for cross-coupling and C–H activation reactions.

Herein, we will explore recent advances and challenges in utilizing DESs as reaction media for cross-coupling and C–H activation reactions, which aim to form molecules by establishing carbon-carbon (C–C) bonds. We will also highlight the advantages and limitations of this approach and shed light on the impact of the physicochemical properties of DESs on the reaction process.

## 2. C–H Activation Reactions

**Scheme 1:**
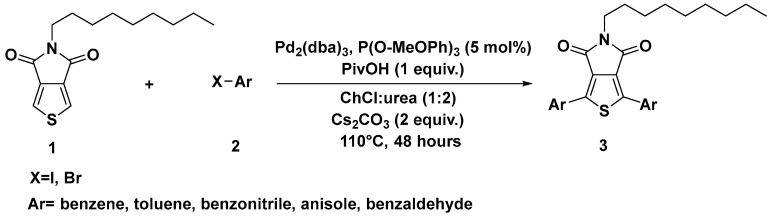
Thiophene−aryl coupling reaction via C–H bond activation in DES.

In 2017, Punzi and coworkers reported, for the first time, the use of DESs in C–H activation reactions to prepare thiophene−aryl derivatives **3** [32]. Hydrophobic DESs worked better than hydrophilic ones, but they decided to choose the hydrophilic DES composed of choline chloride (ChCl) and urea, in a molar ratio of (1:2), as it had a simpler work-up process. In the presence of Pd_2_(dba)_3_ (5 mol%) as the catalyst, P(o-MeOPh)_3_ as the ligand, Cs_2_CO_3_ as the base and pivalic acid (PivOH) (1 equiv.) as additive, the diarylation of 5-octylthieno [3,4-c]pyrrole-4,6-dione (TPD) **1** with aryl iodides **2** successfully occurred, providing high yield of the desired product **3** (Scheme 1). No reaction took place in the absence of the ligand. Electron rich aryl iodides were more reactive than electron poor ones, yielding moderate to good yields. Bromobenzene exhibited less reactivity as a coupling partner, resulting in low yields. The optimized reaction conditions were used to prepare aromatic molecules for push-pull molecular and polymeric semiconductors.

**Scheme 2:**
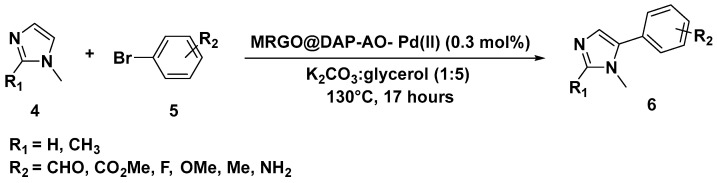
The arylation of imidazoles via C–H bond activation.

Heydari and co-workers described an environmentally safe approach for the arylation of imidazoles with aryl bromides in DESs [33]. Authors synthesized a palladium-based magnetic reduced graphene oxide (MRGO@DAP-AO-Pd(ll)) as a reusable catalyst for C-5 arylation of 1,2-dimethyl-1H-imidazole **4** with 4-bromobenzaldehyde **5**. Various types of choline chloride based DESs were studied. The best solvent for the reaction system was K_2_CO_3_:glycerol (1:5) (Scheme 2). DES can also offer several advantages for this reaction. The high basicity of the solvent can promote the deprotonation of the imidazole compound, which can enhance its reactivity towards the palladium catalyst [34].

Various electron rich and electron deficient aryl halides were used to investigate the limitations of the optimized conditions for the C5-arylation of 1,2-dimethyl-1H-imidazole. C5-arylated products **6** were obtained in good yields using 1-methyl-1H-imidazole with several (hetero)aryl bromides. C5 position of imidazole is more reactive than C2 and C4 position for monoarylation, with only traces of the C2-arylated product detected. The catalyst could be used for seven subsequent runs with a broad substrate scope without significant loss in activity within this protocol.

**Scheme 3:**
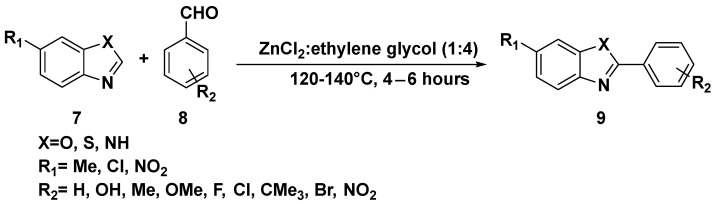
DES–catalyzed arylation of benzoxazoles with aromatic aldehydes.

Recently, Tran and Hang have described DES-catalyzed arylation of benzoxazoles **7** with aromatic aldehydes **8** (Scheme 3) [35]. When using ZnCl_2_:ethylene glycol (1:4) as both the catalyst and solvent, benzoxazoles can be combined with benzaldehyde derivatives to form C2-arylation products **9**. Without exclusion of air, benzoxazoles and aromatic aldehydes, either with electron donating or electron withdrawing groups, yielded the desired products in high to excellent yields. High to excellent yields of the desired products were obtained at 120–140 °C for 4–6 h. In addition, benzothiazole and benzimidazole were compatible with aromatic aldehydes affording good yields of the desired products **9**. Extraction with diethyl ether was performed once the reaction finished. As an interesting side note, DES ZnCl_2_:ethylene glycol (1:4) can be reused up to five times without significant loss in activity. Benzoxazoles arylation has been reported frequently in different solvents. These solvents include H_2_O/diglyme [36], PhCl [37], DMF [38], pivalonitrile [39], and p-xylene [40].

**Scheme 4:**
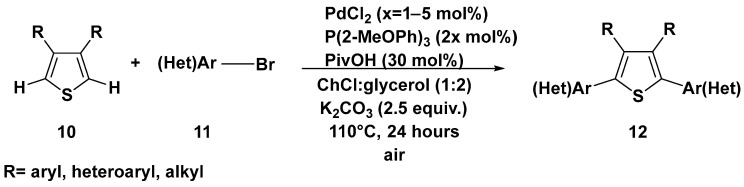
Pd–catalyzed direct arylation of thienyl derivatives with (hetero)aromatic bromides in DESs.

In a recent report, D’Amico et al. demonstrated the use of DES as a sustainable and benign solvent to directly arylate a series of 3,4-disubstituted thiophenes. These thiophenes are well-known for their wide range of applications in optoelectronics, from photovoltaics to semiconductors and electrochromes [41]. ChCl:glycerol (1:2) was chosen as the DES for its high biocompatibility and biodegradability [42] (Scheme 4). A catalytic system consisting of Pd(Cl)_2_ (x = 1–5 mol%), P(2-MeOPh)_3_ (2x mol%) as the ligand, PivOH (30 mol%) as additive, and K_2_CO_3_ (2.5 equiv.) as the base at 110 °C for 24 h could effectively diarylate 3,4-ethylenedioxythiophene **10** with aryl and heteroaryl bromides **11** in moderate-to-high yield of the target product **12**. Air can promote the oxidation of Pd(0) to Pd(II) after the catalytic cycle of C–H activation reaction [43]. This allows palladium to be reused in subsequent cycles of the transformation. As a result of undertaking the reaction under air, there is no need to use inert gas atmospheres and the setup becomes much simpler. This can also reduce the environmental impact of the reaction by eliminating the need to purge the inert gas.

In this strategy, electron poor bromides provided excellent yield even with low catalyst loading (1.0 mol%), while electron rich bromides needed more of the catalyst (5.0 mol%) for a good yield. Ortho-substituted bromides did not affect the reaction negatively, and heteroaromatic bromides, such as 2-bromopyridine, were moderately reactive. Note that simple recrystallization with EtOH or EtOAc is often sufficient for purification.

**Scheme 5:**
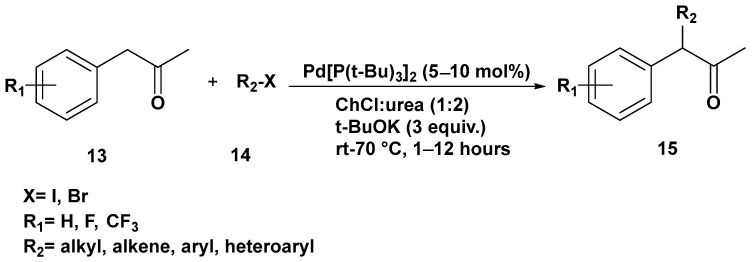
Synthesis of 1-arylpropan-2-ones in DES.

Very recently, Vitale and colleagues reported a new strategy for synthesizing 1-arylpropan-2-ones in DESs under aerobic conditions (Scheme 5) [44]. These compounds are known for their pharmacological activity [45,46]. The group began their study by optimizing the conditions for preparing the enolate intermediate. They achieved this by subjecting 1-phenylpropan-2-one **13** to t-BuOK as the optimum base and ChCl:urea (1:2) as the eutectic mixture at room temperature for 1 h. Alkyls and alkenes **14** with different electronic natures were compatible and generated the desired α-substituted products **15** in high to excellent yields. A series of arylpropan-2-ones with fluorine or CF_3_ at *ortho*-, *meta*-, or *para*- position of aryl ring afforded moderate to high yields.

In this study, the authors arylated 1-phenylpropan-2-one **13** at room temperature for 1 h with t-BuOK (3 equiv.) as the base, and ChCl:urea (1:2) as the solvent. Then, they added iodoaryls or bromoaryls **14** (1.1 equiv.) and Pd[P(t-Bu)_3_]_2_ (5–10 mol%) to the mixtures and heated it at 45–70 °C for 2–12 h. The yield of the products ranged from moderate to excellent, with iodoaryls more reactive than aryl bromides. DESs have shown great potential in activating carbonyl-containing substrates for alpha-arylation. The activation process involves the formation of a hydrogen bond between the carbonyl group of the substrate and the HBD moiety of the DES. This interaction enhances the electrophilicity of the carbonyl group, facilitating the formation of the desired α-substituted product [47,48].

**Scheme 6:**
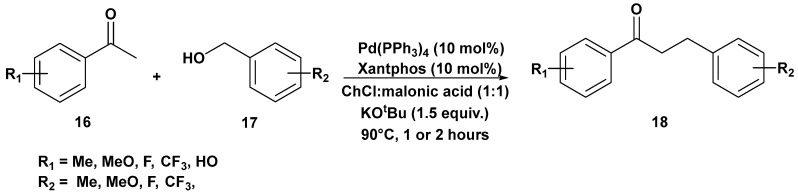
Palladium-catalyzed sp^3^ C–H functionalization of methyl ketones.

Nawaz Khan et al. developed a novel approach for sp^3^-CH functionalization of acetophenones **16** with benzyl alcohols **17** using DES [49]. The optimized conditions include Pd(PPh_3_)_4_/xantphos as the catalyst/ligand combination and KOtBu (1.5 equiv.) as the base. Among the DESs used, ChCl:malonic acid (1:1) was found to be the most effective solvent, producing high yields of the α,β-saturated ketones **18** (Scheme 6). The use of ChCl−oxalic acid (1:1) as the DES mixture also led to the desired product in good yields. A variety of electron donating and electron withdrawing groups were tolerated on acetophenone and benzyl alcohol to isolate the desired α,β-saturated ketones in good to high yields. By using the ChCl-based DESs as alternatives to toluene, the acidic pH of the DESs neutralized the reaction medium from strongly basic to slightly basic. This adjustment reduced the likelihood of ketone reduction into alcohols and the formation of side products. The mechanism of DES involves hydrogen bonding, activating benzyl alcohol before dehydrogenation catalyzed by the palladium complex. The DES medium enhances electrophilicity by interacting with the oxygen atom of the ketone group, promoting the nucleophilic attack to form an α-alkylated saturated ketone **18**.

**Scheme 7:**
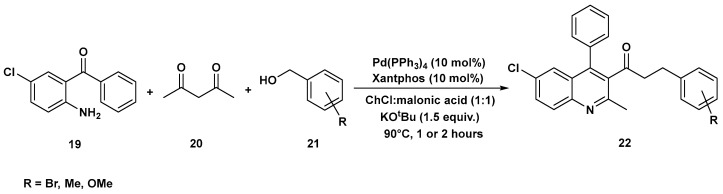
Friedländer reaction and palladium-catalyzed, sp^3^ C–H functionalization of methyl ketones.

The Friedländer reaction between ketone **19** and acetylacetone **20** suggests that DES acted as both the solvent and catalyst. The DES formed hydrogen bonds with the reagents, facilitating the reaction, and the formation of a chalcone intermediate was observed [50]. The reaction involved an N-heterocyclic ketone that was 4-α-alkylated with benzyl alcohols **21**, resulting in a good yield of the desired product **22**. Various substituents on benzyl alcohol were compatible and steric hindrance had no effect on the reaction yield (Scheme 7). DES was reused for five consecutive cycles, with no observable detrimental effects on yields.

**Scheme 8:**
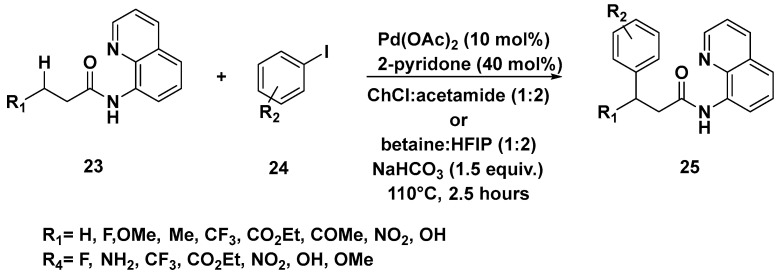
Arylation of amide derived from 8-aminoquinoline.

Marset recently described a strategy for the free-silver-mediated Csp^3^–H functionalization of unactivated 8-aminoquinoline amides using palladium catalysts in a DES [51]. He established the conditions for functionalizing the amide derived from 8-aminoquinoline **23** with aryl iodides **24**. The conditions involved Pd(OAc)_2_ (10 mol%) as the catalyst, NaHCO_3_ (1.5 equiv.) as the base, and 2-pyridone (40 mol%) as the ligand. The reaction was carried out in betaine (choline derivative):hexafluoroisopropanol (HFIP) (1:2) as the solvent at 110 °C for 2.5 h, resulting in a good to high yields of the desired products **25**. Additionally, ChCl:acetamide (1:2) also proved effective among the various DESs evaluated, providing the desired products in good yields (Scheme 8).

**Scheme 9:**
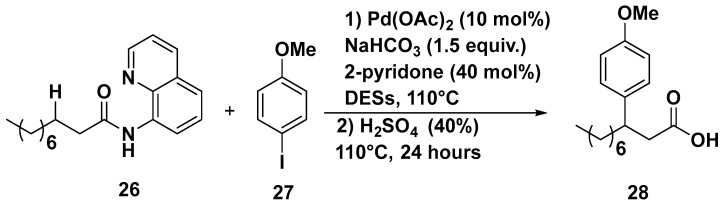
One-pot directing group removal.

The protocol was efficient with aryl iodides that had both electron withdrawing and electron donating groups in betaine:HFIP (1:2) and ChCl:acetamide (1:2) DESs, resulting in good to excellent yields. Betaine:HFIP provided higher yields. However, ortho-substituted aryl iodides had drastic results due to steric hindrance. In ChCl:acetamide (1:2), shorter alkyl-chain lengths resulted in marginal yields, while full conversions were obtained in betaine:HFIP (1:2). Furthermore, a one-pot directing group removal was presented by quenching the reaction with 40% aqueous sulfuric acid [52] and heating the mixture at 110 °C for 24 h, affording **28** in a good yield (Scheme 9). Although the reusability of DESs with the catalyst may be limited to no more than two cycles, DESs can facilitate the recycling of transition-metal catalysts in multiple reaction processes. This can result in a substantial enhancement of the overall turnover number of reactions [53,54].

**Scheme 10:**
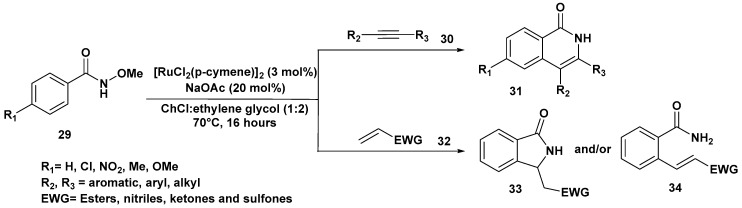
C–H activation reaction using disubstituted alkynes and electron poor olefins.

Recently, in 2022, a research team led by González–Gallardo published a study on a highly effective method for C–H activation using a ruthenium catalyst in DESs [55]. The optimized conditions for the reaction involved 3 mol% [RuCl_2_(p-cymene)]_2_ as the catalyst, and 20 mol% NaOAc as an additive, at 70 °C for 16 h. After examining various DESs, ChCl:ethylene glycol (1:2) was the most effective solvent. Choline chloride-based DESs are highly polar solvents that can increase reaction rates and stabilize reactive intermediates, making them effective for polar reactions [56]. Electron withdrawing substituents on the *para* position of N-methoxybenzamide **29** reacted with internal alkynes with aromatic substituents **30** and favored the desired isoquinolones with high yields **31** (Scheme 10). The DES can be reused for up to three times without any significant loss in yield. Isoquinolone derivatives have displayed various pharmacological and biological effects, including antitumor [57], anti-inflammatory [58], and antihypertensive activities [59].

The C–H activation reaction with N-methoxybenzamide **29** proved effective for electron poor olefins **32** under identical conditions. High yields were achieved when utilizing olefins containing electron withdrawing groups. Interestingly, exceptional Michael acceptor reagents, such as vinyl ketone and phenyl–vinyl, sulfone-generated cyclic products **33,** or a mixture of acyclic and cyclic products **33** and **34,** had excellent yields (Scheme 10).

**Scheme 11:**
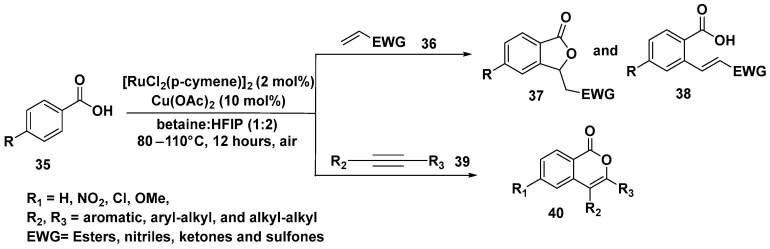
C–H activation reaction using electron poor olefins and disubstituted alkynes with benzoic acid derivatives.

To assess the flexibility of this approach, Cu(OAc)_2_ (10 mol%) was used instead of NaOAC, and betaine:HFIP (1:2) was chosen as the solvent among various DESs tested. A variety of olefins **36,** containing electron withdrawing groups, reacted with benzoic acid **35**, leading to high yields of the products **37** and **38**. Electron withdrawing substituents at the para position in the carboxylic acid **35** favored the reaction, while electron donating groups showed lower reactivity. Experiments were conducted using various disubstituted alkynes **39** to create isocoumarin derivatives **40**. Isocoumarin scaffolds are a group of natural products that are biologically, structurally, pharmacologically fascinating, and commonly used in drug discovery, pharmaceutical and medical chemistry [60,61]. Good yields were obtained when carboxylic acids were subjected to internal alkynes with aromatic, aryl–alkyl, and alkyl–alkyl substituents **39** (Scheme 11).

**Scheme 12:**
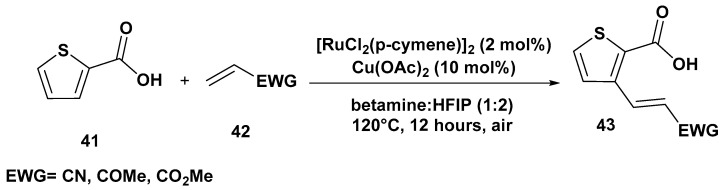
C–H activation reaction using electron poor olefins with 2-thiophenecarboxylic acid.

Successful synthesis of heterocycles commonly found in drug candidates [62] was achieved by reacting 2-thiophenecarboxylic acid **41** with various olefins **42** containing electron withdrawing groups. Every reaction produced moderate to good yields of exclusively acyclic derivatives **43**. To demonstrate the method’s applicability, the reaction was scaled up to the gram level, resulting in excellent yield of the desired product through precipitation with a small amount of water (Scheme 12). The reaction was conducted under air conditions, air can act as an oxidant to make the reaction more cost-effective and safer than other commonly used oxidants, such as peroxides or molecular oxygen.

**Scheme 13:**
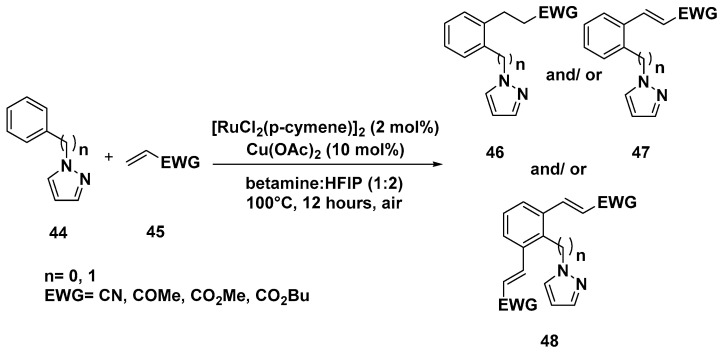
C–H activation reaction using electron poor olefins with 1-arylpyrazole derivatives.

Under the previously mentioned conditions, and to broaden the range of substrates for this transformation, several aryl pyrazole derivatives **44** and electron poor olefins **45** were tested, resulting in moderate to good yields. Interestingly, the use of methyl–vinyl ketone as a substrate, led to the production of both saturated **46** and unsaturated compounds **47** in similar proportions. Additionally, in some instances, a double addition product to 1-phenylpyrazole **48** was observed (Scheme 13). Certain arylpyrazoles have exhibited noteworthy pharmacological activities [63].

## 3. Cross-Coupling Reactions

**Scheme 14:**
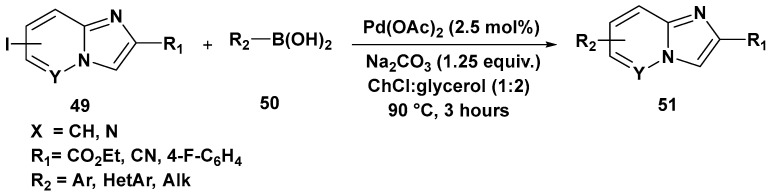
Suzuki–Miyaura cross-coupling reactions applied to imidazo-fused heterocycles.

In 2018, Delaye et al. reported the first Suzuki–Miyaura cross-coupling in a natural DES (NaDES) [64] to arylate biologically active imidazo [1,2-a]pyridines and imidazo [1,2-b]pyridazines **49** [65,66]. C3-arylation reaction of ethyl 3-iodo-imidazo [1,2-a]pyridine-2-carboxylate **49** with phenylboronic acid **50** as the arylating agent was performed (Scheme 14). The reaction was catalyzed by Pd(OAc)_2_ (2.5 mol%) and Na_2_CO_3_ (1.25 equiv.) as the base was used. The reaction was carried out in mannose:DMU (3:7) solvent with oil-bath heating at 90 °C overnight without the exclusion of air. The desired product **51** was isolated in excellent yield. Then, various DESs were tested. ChCl:glycerol (1:2) was a good solvent, producing the desired product in high yield within just 1–2 h. Two years later, Bin Sun et al. reported a highly selective direct C–H alkylation of imidazo [1,2-a]pyridines using organic photoredox catalysis in polar solvents, such as DMSO, enabling mild conditions [67]. Combining this with DESs can enhance sustainability and promote green chemistry principles.

The study investigated the effectiveness of mannose:DMU (3:7) and ChCh:glycerol (1:2) in functionalizing iodinated compounds **49** with PhB(OH)_2_ in the imidazo [1,2-a]pyridine series. ChCh:glycerol (1:2) was more effective, and the reaction time varied based on the position of the iodine substituent. The strategy demonstrated high tolerance to arylboronic acids and exhibited good reusability of the catalyst for up to three runs, with a slight reduction in yield observed in the fourth and fifth runs.

**Scheme 15:**
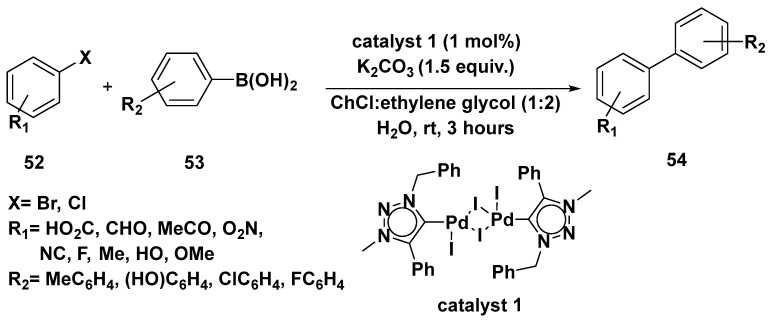
Suzuki–Miyaura cross-coupling reaction in DES.

Marset et al. recently synthesized a σ-donor mesoionic carbene ligand for cross-coupling transformations in DESs [68]. They optimized the reaction conditions using N-heterocyclic carbene (NHC)-Pd (**catalyst 1**) (1 mol%) as the catalyst, K_2_CO_3_ (1.5 equiv.) as the base, and ChCl:ethylene glycol (1:2) DES with 10 equivalent of water at room temperature for 3 h. Surprisingly, adding 20 equivalent of water resulted in a decrease in reaction yield (Scheme 15). Small amounts of water can improve solubility and reaction rates [69]. Excessive water addition can have the opposite effect, reducing solubility and reaction efficiency. Yu Chen et al. found that the precise control of water addition to ChCl:ethylene glycol (1:2) is crucial to maintain solvent stability [70]. Several aryl bromides and chlorides **52** were successfully subjected to phenylboronic acid **53**, resulting in moderate to excellent yields. Aryl bromides were slightly more effective than aryl chlorides; 4′-bromoacetophenone reacted smoothly with phenylboronic acid containing various substituents, affording the desired products **54** in excellent yields.

**Scheme 16:**
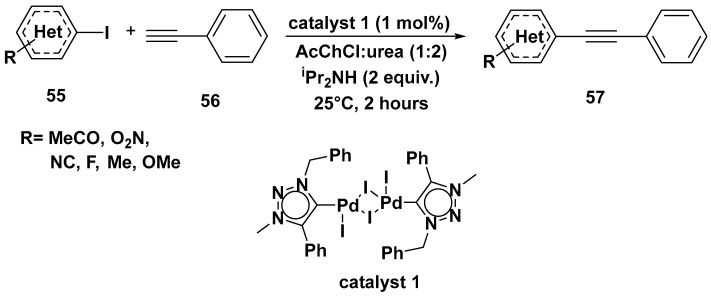
Sonogashira coupling reaction in DES.

Among the diverse DESs tested, Acetylcholine chloride (AcChCl):urea (1:2) was found to be the preferred solvent for Sonogashira reaction. AcChCl:urea (1:2) has a high polarity and can dissolve both the palladium catalyst and materials, such as halides, efficiently. It has a low viscosity, which allows for better mixing and faster reaction times. Various aryl iodides **55** were reacted with phenylacetylene **56** in the same catalyst with the base ^i^Pr_2_NH for two hours at room temperature, resulting in moderate to excellent yields of the desired products **57**. Electron-poor aryl iodides generally had better yields compared to neutral or electron-rich aryl iodides. In some cases, moderate yields could be enhanced by increasing the reaction temperature or extending the reaction time (Scheme 16).

**Scheme 17:**
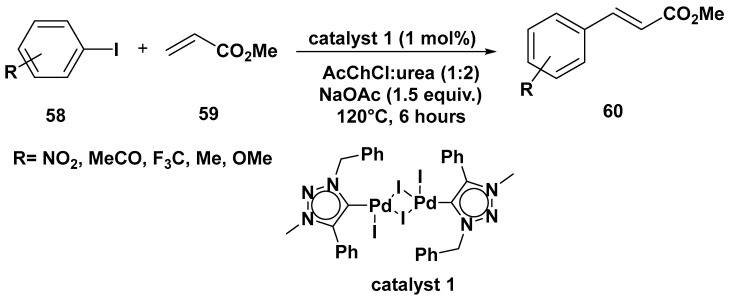
Heck–Mizoroki cross-coupling reaction in DES.

The best DES for Heck coupling with **catalyst 1** was chosen as AcChCl:urea (1:2). The reaction was conducted at 120 °C for 6 h, with the base NaOAc. Methyl acrylate **59** reacted well with various aryl iodides **58**, giving satisfactory to excellent yields of the desired products **60**. However, electron-rich aryl iodides required a longer reaction time (16 h) to achieve good to excellent yields. Notably, 4′-bromoacetophenone and 4′-chloroacetophenone were not compatible with this transformation (Scheme 17).

**Scheme 18:**
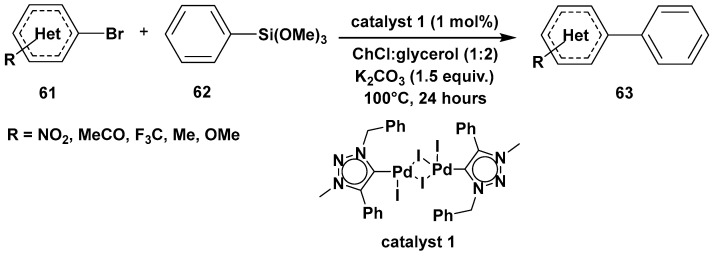
Stille cross-coupling reaction in DES.

The Hiyama reaction was conducted utilizing K_2_CO_3_ (1.5 equiv.) as the base and a eutectic mixture of ChCl:glycerol (1:2) at 100 °C for 24 h. The reaction involved aryl bromides **61** with phenyltrimethoxysilane **62**. Both electron-deficient and electron-rich aryl bromides exhibited excellent reactivity, providing the desired product **63** in good to excellent yields. Notably, heteroaryl bromides performed exceptionally well and produced excellent yields of the desired product (Scheme 18).

**Scheme 19:**
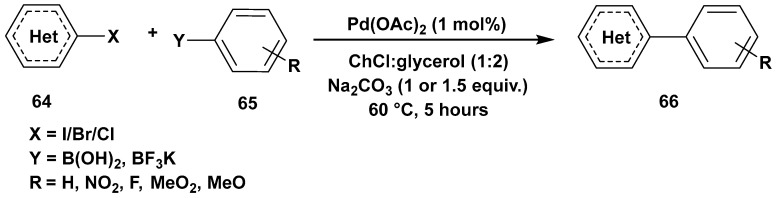
Suzuki–Miyaura couplings using aryltrifluoroborates as effective partners in DESs.

A recent report by Capriati and colleagues introduced an efficient strategy for Suzuki–Miyaura couplings using aryltrifluoroborates in DESs [71]. Chlorobenzene **64** was coupled with phenylboronic acid **65** using Pd(OAc)_2_ as the catalyst (1 mol%) and Na_2_CO_3_ (1 or 1.5 equiv.) as the base in ChCl:glycerol (1:2) eutectic mixture at 60 °C for 5 h, resulting in a quantitative yield of the desired product **66** (Scheme 19). The study tested various DESs, but ChCl:glycerol and ChCl:urea (1:2) were found to be the most effective solvents. Promising results were obtained in the reaction using aryl halides **64** with electron donating or electron withdrawing groups, along with aryltrifluoroborates **65** or arylboronic acids **65**. Additionally, heteroaryl halides such as indole, pyridine, and thiophene were also successful in the cross-coupling reactions with aryltrifluoroborates, yielding desired products **66** in good yields. The use of arylboronic acids with aryl halides in Pd-catalyzed Suzuki–Miyaura reactions are described in biomass-derived solvents [72,73,74,75,76] and ILs [77,78,79,80]. However, harsh heating conditions and soluble phosphines were typically required. The use of DESs as a reaction medium for these reactions is a recent, more sustainable development.

Aryl halides with electron-donating or electron-withdrawing groups reacted with phenylboronic acid, leading to target compounds in good to excellent yields. Substituted aryltrifluoroborates were also compatible affording moderate to high yields. This methodology was successfully extended to the synthesis of non-steroidal anti-inflammatory drugs, Felbinac and Diflunisal [81]. This protocol was also compatible for the synthesis of terphenyl. The DES, when used with a catalyst and base, can be reused for four to six subsequent reactions.

**Scheme 20:**
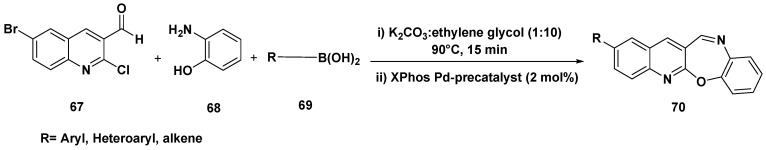
Pd-Precatalyst mediated Suzuki-Miyaura reaction in DES.

Recently, Nawaz Khan and coworkers described a novel method for synthesizing benzo[2,3][1,4]oxazepino [7,6-b]quinolines [82], known to exhibit antidepressant, antagonist, anti-cancer, and squalene synthase inhibitory properties [83]. A cyclization reaction between 6-bromo-2-chloroquinoline-3-caboxaldehyde **67** and 2-aminophenol **68** in K_2_CO_3_:ethylene glycol (1:10) for 15 min at 90 °C, led to isolate benzo[2,3][1,4]oxazepino [7,6-b]quinoline. K_2_CO_3_:ethylene glycol (1:10), selected from a large range of DESs, exhibited high selectivity due to strong hydrogen bondings (Scheme 20). Following this, the benzo[2,3][1,4]oxazepino [7,6-b]quinoline was arylated by Suzuki-Miyaura coupling with phenyl boronic acid **69** in the presence of 2 mol% XPhosPdG2 to provide high yields of benzoxazepines **70**. Several substituted phenylboronic acids with electron-donating or electron-withdrawing groups were found to be highly tolerated and provided high yields. Heterocyclic boronic acids, including indole, pyridine, thiophene, pyrazole, and bulky hindered dibenzothienyl groups, generated high yields.

**Scheme 21:**
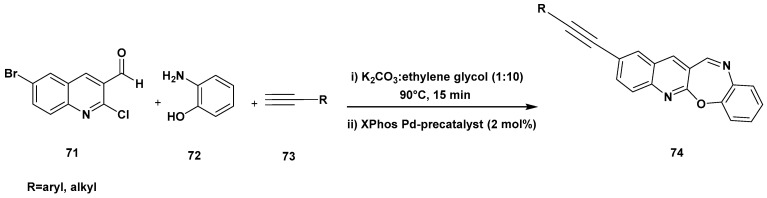
Pd-Precatalyst mediated Sonogashira reaction in DES.

Under the same optimized conditions, Sonogashira reaction of benzo[2,3][1,4]oxazepino [7,6-b]quinoline with various aliphatic terminal alkynes **73** resulted in high yields of the desired products **74**. Excellent yields were obtained with a variety of electron-poor and electron-rich aromatic alkynes (Scheme 21).

**Scheme 22:**
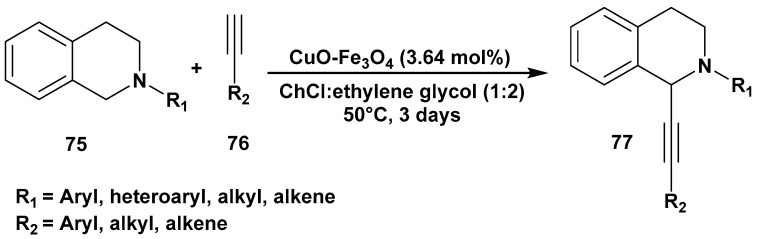
Cross-dehydrogenative Sonogashira coupling reaction using copper oxide impregnated on magnetite in DESs.

Ramón and co-workers reported a cross-dehydrogenative coupling reaction in a DES (Scheme 22) [84]. 2-(4-Fluorophenyl)-1,2,3,4-tetrahydroisoquinoline **75** and phenylacetylene **76** were employed as model substrates in the presence of copper impregnated inthe magnetite catalyst CuO-Fe_3_O_4_ (3.64 mol%) at 50 °C for 3 days. ChCl:ethylene glycol (1:2) was chosen as the best DES among others tested, and an excellent yield of the desired product **77** was obtained. A correlation between eutectic mixtures conductivity and reaction yields was observed, with increased yields being significantly affected by conductivity. The high conductivity of the eutectic mixture facilitates electron transfer and enhances the interactions between the reactants and the catalyst, leading to a higher reaction rate. In a highly conductive solvent, the molecules are able to move more freely and transfer electrons more efficiently [85].

The synthesis of tetrahydroisoquinolines has gained much attention due to their biological and pharmaceutical properties, including anticancer [86,87] anticonvulsant [88], enzyme inhibition [89], receptor-ligand binding [90], and therapeutic efficacy [91]. In this study, the nitrogen atom of tetrahydroisoquinoline was shielded using either electron-rich or electron-poor aryls. The effect of electron donating and electron withdrawing groups on phenylacetylene’s ring was examined. Excellent to reasonable yields were obtained. Additionally, the recyclability of CuO-Fe_3_O_4_ and DES as a catalyst-solvent combination was investigated. The catalyst-solvent system can be recycled for ten consecutive uses without any reduction in activity.

**Scheme 23:**
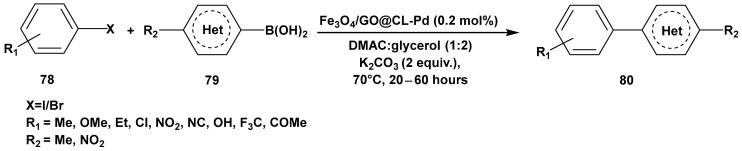
Pd–catalyzed Suzuki–Miyaura coupling reaction in eutectic mixture.

An approach for the preparation of Fe_3_O_4_/GO@CL-Pd catalyst was disclosed by Shekaari group to test its performance in synthesizing biphenyls **80** via Suzuki–Miyaura coupling in eutectic mixtures [92]. A small amount of Fe_3_O_4_/GO@CL-Pd (0.2 mol%) as the catalyst, and K_2_CO_3_ (2 equiv.) as the base, were used for the reaction between 4-iodoanisole **78** and phenylboronic acid **79** at 70 °C for 20–60 min (Scheme 23). Different hydrophilic and hydrophobic DESs were tested, but dimethylacetamide (DMAC):glyceol (1:2) proved to be the most effective, resulting in a quantitative yield of the biaryl compound. The use of hydrophilic DESs likely played a role in facilitating the dispersion of the catalyst, which enhanced catalytic activity [93]. Additionally, hydrophilic DESs simplified the reaction’s workup [94].

Phenylboronic acid is highly compatible with both electron-poor and electron-rich aryl bromides and iodides for producing desired products. Although aryl iodides are slightly more reactive than aryl bromides. The reactivity of arylboronic acids of different nature were found to be high regarding aryl halides. It was also demonstrated that the DES is reusable with catalyst, as it demonstrated stable activity for five consecutive runs.

**Scheme 24:**
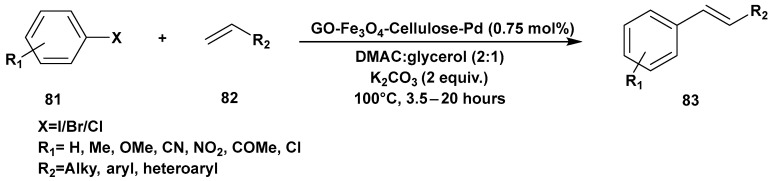
Heck coupling reaction in DES.

Karimi and coworkers recently reported a pathway for Heck and Sonogashira-coupling reactions in DESs [95]. A GO-Fe_3_O_4_-Cellulose-Pd (0.75 mol%) catalyst was used, along with K_2_CO_3_ (2 equiv.) as a base at 100 °C in the DES, composed of DMAC:glycerol (1:2) as the solvent. In a comparison of fifteen DESs, the hydrophilic DES composed of dimethyl–ammonium–chloride and glycerol demonstrated superior performance. A hydrophilic catalyst is likely to enhance its catalytic efficiency in hydrophilic DESs through increased dispersion [93]. Various electron-deficient and electron-rich aryl halides **81** were smoothly coupled to aliphatic and aromatic alkenes **82**, resulting in high to quantitative yields of the products **83** (Scheme 24).

**Scheme 25:**
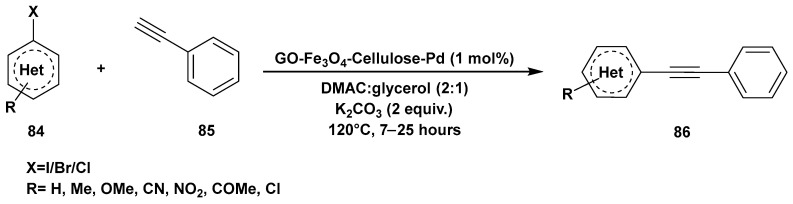
Sonogashira cross-coupling reaction in DES.

The hydrophilic DES with the catalyst proved to be highly effective in successfully coupling various aryl halides **84** via Sonogashira coupling to phenylacetylene **85** under identical conditions (Scheme 25). This approach yielded exceptional yields of the desired product **86**, demonstrating its efficacy. Following the reaction, ethyl acetate and water were used to extract the products and separate the organic phase. Catalyst and DES were recovered from the aqueous phase and found to be reusable for eight subsequent reactions. This finding provides evidence for the advantages of heterogeneous catalysts, such as simple work-up procedures and straightforward catalyst recycling, as previously reported [96,97].

**Scheme 26:**
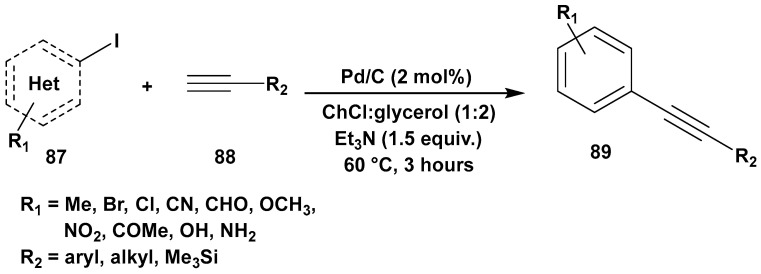
Palladium-catalyzed Sonogashira cross-coupling reaction in DESs.

Salomone’s group has developed a new ligand-free Sonogashira cross-coupling reaction using a recycling DES as a reaction medium [98]. The study used Pd/C (2 mol%) as a heterogeneous catalyst combined with Et_3_N as the base. ChCl:glycerol (1:2) provides a strong and extended hydrogen-bonding network. The use of DESs is paramount in optimizing the effectiveness of heterogeneously Pd-catalyzed cross-coupling reactions [99]. Aryl iodides **87** possessing methyl- or electron-attracting groups significantly increased the yield of the desired products **89** when reacting with phenylacetylene **88** (Scheme 26). Electron-poor aryl iodides were found to be more reactive than electron-rich ones. Interestingly, heteroaryl iodides, such as pyridine and thiophene, produced quantitative yields of the desired heterocyclic compounds when coupled with various types of alkynes. The DES can be used with Pd/C for four runs without a significant loss in activity in the coupling reaction between 1-chloro-3-iodobenzene and phenylacetylene.

**Scheme 27:**
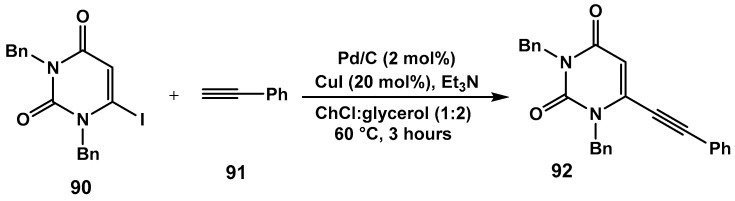
Sonogashira coupling reaction of 6-iodouracil with phenylacetylene in DES.

It’s important to mention that adding CuI (20 mol%) to the latter conditions enabled the Sonogashira coupling reaction of 6-iodouracil **90** with phenylacetylene **91** to be compatible with this approach. As a result, the desired 6-alkynylated uracil **92** was obtained in a good yield (Scheme 27). Uracil derivatives are highly favored molecular frameworks in drug discovery due to their diverse range of activities. They have been researched for their potential therapeutic use in treating viral infections, cancer, diabetic, thyroid and autosomal recessive disorders [100,101].

**Scheme 28:**
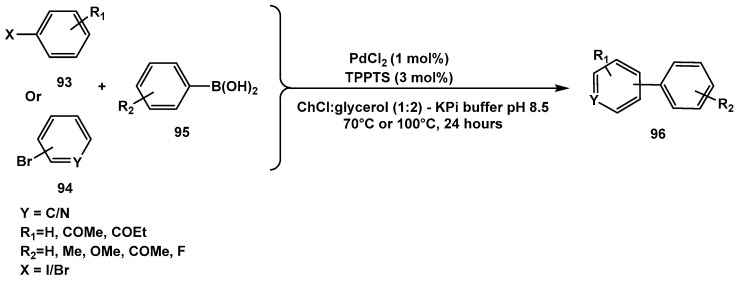
Cascade one-pot synthesis of enantiomerically pure biaryl ketones in DESs.

Groger et al. reported a novel protocol for the synthesis of biaryls using DESs [102]. To model the reaction, they used 4′-bromoacetophenone **93** and phenylboronic acid **95** as substrates and a eutectic mixture of ChCl:glycerol (1:2) with a potassium phosphate buffer (KPi) at pH 8.5. While various DESs were evaluated, this specific eutectic mixture with KPi was ultimately chosen for the Suzuki–coupling reaction, which was catalyzed by PdCl_2_ (1 mol%)/TPPTS (3 mol%) as the catalyst/ligand combination. The buffer played a crucial role in maintaining the pH of the reaction mixture at an optimal level [103]. The Suzuki–coupling reaction requires a slightly basic environment for the reaction to occur, and the buffer would have helped to maintain this pH range [104,105]. A pH 8.5 KPi, buffer, used to efficiently carry out the reaction, resulted in a quantitative yield of 4′-phenylacetophenone **96** (Scheme 28).

To enhance the production of the target compounds from iodo and bromoacetophenones **93** with aryl boronic acids **95**, the reaction conditions were systematically refined for optimal yields. The substrate scope was expanded to include bromopyridines **94**, which reacted efficiently with aryl boronic acids to provide the expected products in quantitative yields.

**Scheme 29:**
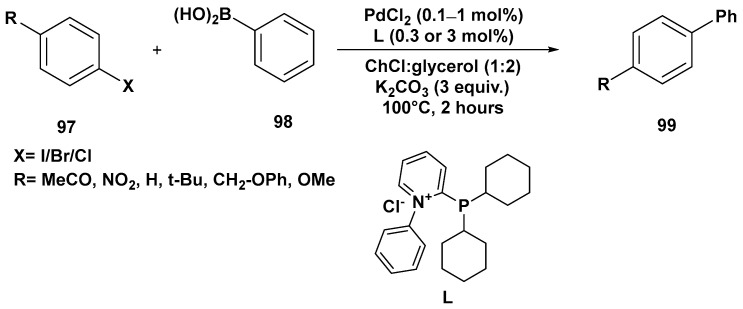
Palladium catalyzed Suzuki cross-coupling reaction in DES.

Ramón’s group reported an approach for cross-coupling reactions in DESs [53]. A palladium precursor, PdCl_2_ (1 mol%), was used in combination with **pyridiniophosphine** (**L**) (3 mol%) as a ligand at 100 °C for 2 h. The best results were obtained with the eutectic mixture composed of choline chloride combined with either ethylene glycol or glycerol (Scheme 29). The yields were similar for both solvents, but the study used choline chloride:glycerol (1:2) due to its sustainability [106]. By utilizing phenylboronic acid **98** with an array of para-substituted aryl halides **97**, encompassing both iodides and bromides, the desired products **99** were isolated in excellent to quantitative yields. Aryl halides with electron withdrawing groups showed slightly higher efficacy compared to those with electron donating groups.

**Scheme 30:**
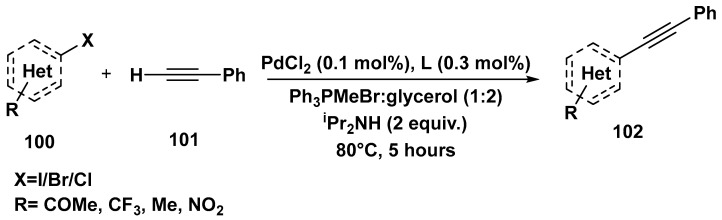
Palladium catalyzed Sonogashira cross-coupling reaction in DES.

The Sonogashira-coupling reaction between 4-iodoacetophenone **100** and phenylacetylene **101** provided a quantitative yield by utilizing ^i^Pr_2_NH (2 equiv.) as the base in Ph_3_PMeBr:glycerol (1:2) for the eutectic solvent. Although electron-poor aryl iodides showed better reactivity, good yields were also obtained from electron-rich ones. Steric hindrance had a negative impact on the yield of the reaction (Scheme 30). Notably, this method was effective with heteroaryl bromides and iodides, such as pyridine and thiophene, providing good to excellent yields of the desired products **102**.

**Scheme 31:**
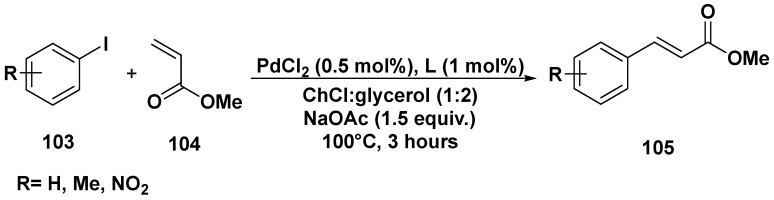
Heck cross-coupling reaction in DES.

A Heck cross-coupling reaction was performed to explore the limitations of this approach. In this study, Pd(Cl)_2_ (0.5 mol%) was used as the catalyst, pyridiniophosphine L (1 mol%) as the ligand, and NaOAc as the base, in ChCl:glycerol (1:2) as the reaction medium. By using 1-iodo-4-nitrobenzene **103** and methyl acrylate **104**, 89% of the desired product **105** was obtained (Scheme 31). A variety of electron-rich and electron-poor aryl iodides were subjected to methyl acrylate, producing moderate-to-good yields. It should be noted, that steric hindrance adversely affected the yield of the reaction.

Research has been conducted to examine the possibility of recycling the catalyst and solvents in Suzuki and Sonogashira reactions [107,108]. Results revealed that both the catalyst and solvents could be reused up to five times without significant loss in catalytic activity. Pd-catalyst structure was also studied using titration, NMR, and DFT methods, to confirm phosphine‘s coordination to palladium. These investigations provided insights into the coordination properties of DES-compatible, cationic-phosphine ligands, allowing for a more comprehensive understanding of the reaction mechanism. Overall, the recycling of catalysts and DESs can contribute to a more sustainable and cost-effective approach to chemical processes.

**Scheme 32:**
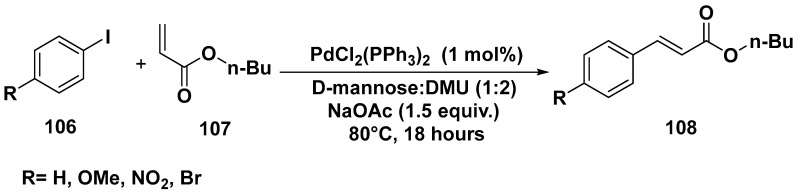
Heck cross-coupling reaction in eutectic mixture.

Konig and Ilgen investigated whether sugar melts might replace polar solvent [109] for the Heck cross-coupling reaction [110]. This study used a homogeneous PdCl_2_(PPh_3_)_2_ catalyst with NaOAc (1.5 equiv.) as the base at 85 °C for five hours under ultrasound, in a D-mannose:dimethylurea (DMU) (1:2) mixture. Different substituted iodobenzenes **106** and n-butyl acrylate **107** showed excellent to good yields of the reaction products **108** (Scheme 32). As compared to conventional solvents and ionic liquids, sugar melts achieved good yields at lower temperatures with ultrasound agitation [111]. In spite of the slightly high viscosity, D-mannose:DMU (1:2) mixtures showed promising solvent properties for chemical transformations [112].

**Scheme 33:**
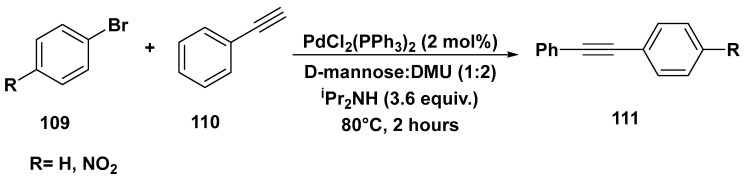
Sonogashira cross-coupling reactions in a eutectic mixture.

Choosing the right solvent polarity is crucial to the success of Sonogashira cross-coupling reactions. By using deep eutectic mixtures as reaction media, in combination with a homogeneous catalyst PdCl_2_(PPh_3_)_2_ (2 mol%) and ^i^Pr_2_NH (3.6 equiv.) as the base, the reaction can proceed smoothly. Through extensive exploration of eutectic mixtures, it was observed that D-mannose:DMU (3:7) exhibited the highest polarity, thus proving to be the ideal reaction medium. Under the optimized conditions, the coupling of phenylacetylene **110** with 1-bromo-4-nitrobenzene and bromobenzene **109** resulted in the desired products **111** in good yields (Scheme 33). Additionally, the method can be used to synthesize natural products that contain acetylene functionalities. The importance of these applications have been noted in previous works: references [113,114].

**Scheme 34:**
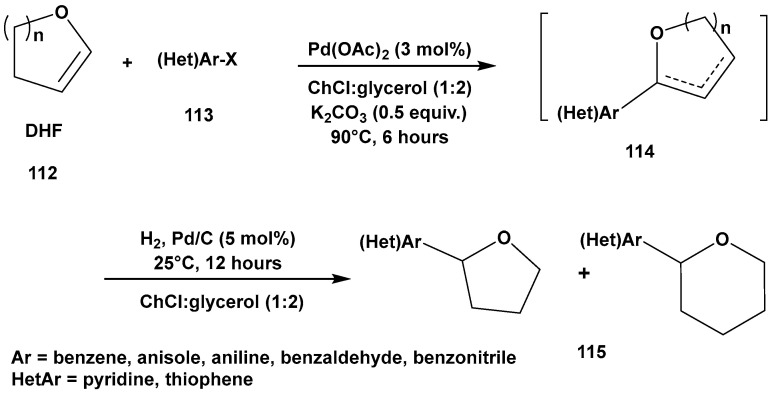
One-pot reductive Mizoroki–Heck reaction in DES.

Dilauro et al. recently reported a one-pot reductive Mizoroki–Heck reaction using a free-ligand Pd-catalyst to synthesize functionalized oxygen heterocycles [115]. The reaction involved 2,3-dihydrofuran **112** and 3-bromopyridine **113** in air, with a mixture of ChCl:glycerol (1:2) as the solvent (Scheme 34). A regioisomeric mixture **114** was produced in a quantitative yield by using Pd(OAc)_2_ (3 mol%) as the catalyst and K_2_CO_3_ (0.5 equiv.) as the base at 60 °C for 6 h. After 12 h of stirring, the desired hydrogenated product **115** was isolated in heterogeneous conditions with a quantitative yield from the regioisomeric mixture, using Pd/C (5 mol%) as a catalyst. The ineffective recycle of the solvent/catalyst is due to their modification during the reaction process, leading to a decreased yield and increased viscosity. The precipitation of Pd-black indicates that the catalyst has been modified and is no longer efficient, making it impossible to recycle.

The authors investigated fifteen final product examples using 2,3-dihydrofuran (DHF) **112**. Effective substrates included aryl iodides bearing electron-donating or electron-withdrawing groups, which afforded the desired products with a high to excellent yield. In addition, the viable substrate of the five-membered heterocycle 2-iodothiophene was utilized. The protocol was also successful in synthesizing 2-(hetero)aryl 3,4-dihydro-2H-pyran (THP) derivatives, with different electron-poor and electron-rich iodoaryls, resulting in moderate to good yields. This strategy was surveyed for the synthesis of tetrahydrofuran (THF) derivatives on a 2 g scale. These derivatives are known to be potent inhibitors of Kv1.2 channel and have been suggested for stroke treatment, as anti-depressants, and for their lipid-lowering activity [116,117,118,119].

**Scheme 35:**
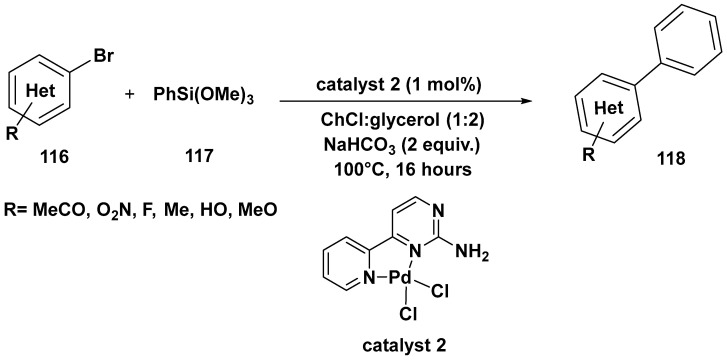
Hiyama cross-coupling reaction in DES.

Saavedra and colleagues have recently investigated the use of a nitrogen-based bipyridine–palladium derivative as a catalyst for cross-coupling reactions in a DES [120]. Although phosphine ligands are commonly used in palladium-catalyzed cross-coupling reactions, nitrogen-based ligands are cheaper and have received less attention [121,122,123]. Bipyridine-palladium **catalyst 2** (1 mol%) [124] was employed in combination with NaHCO_3_ as the base. The reaction was carried out in ChCl:glycerol (1:2), which was selected as the eutectic mixture due to its superior performance compared to other DESs tested. The reaction was conducted at a temperature of 100 °C for 16 h. The exceptional efficiency of ChCl:glycerol was attributed to the strong interactions between the catalyst and the eutectic mixture, which were facilitated by the free-amino group that was present in the catalyst. These interactions resulted in the formation of hydrogen-bond networks with the DES, thereby enhancing the catalytic efficiency. The outcome of the reaction provided excellent isolated yields when trimethoxyphenylsilane **117** was reacted with various electron-rich and electron-poor aryl bromides **116**. Moreover, the reaction provided good yields of the desired products using heteroaryl bromides **118** (Scheme 35).

**Scheme 36:**
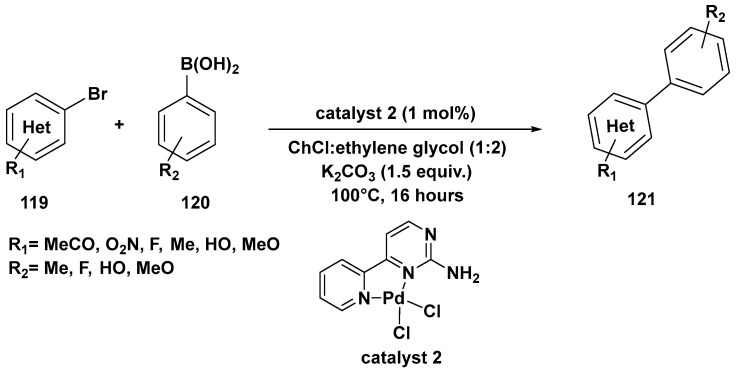
Suzuki–Miyaura cross-coupling reaction in deep eutectic mixture.

Suzuki–Miyaura reaction conditions were refined by optimizing the ase and solvent choice. The same **catalyst 2** was used. ChCl and ethylene glycol, in a 1:2 ratios, was the optimal solvent, and K_2_CO_3_ was the best base. The reaction was carried out at 100 °C for 4 h. It was observed that aryl bromides **119** bearing electron withdrawing groups exhibited higher yields than those bearing electron donating groups when reacting with substituted phenylboronic acid **120**. Heteroaryl bromides resulted in only moderate yields of the desired products **121**. Different substituted boronic acids reacted with 4-bromoacetophenone, with better yields achieved when using electron-rich phenylboronic acids (Scheme 36).

**Scheme 37:**
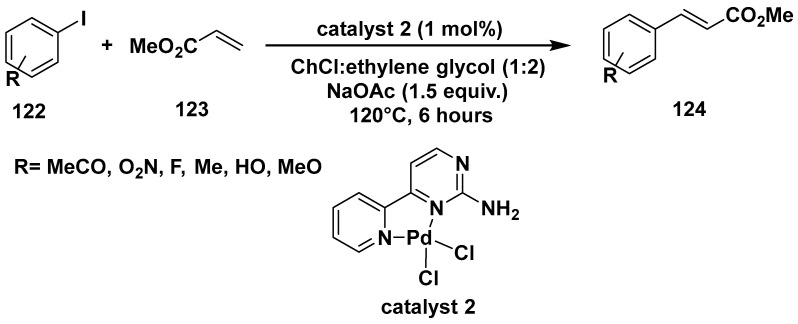
Heck–Mizoroki cross-coupling reaction in DES.

The Heck–Mizoroki reaction was conducted at 120 °C for 6 h using NaOAc (1.5 equiv.), while the Suzuki–Miyaura conditions were maintained. A range of excellent to good yields of the target products **124** were observed when methyl acrylate **123** was reacting with aryl iodides **122** with *para*-electron-withdrawing groups. Additionally, aryl iodides bearing electron-donating groups showed a similar effectiveness, except for the 2-methyl and 4-methyl derivatives, which yielded slightly lower yields (Scheme 37).

**Scheme 38:**
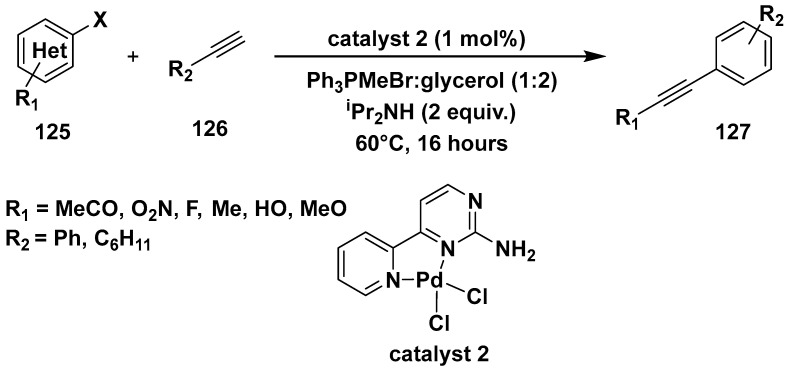
Sonogashira cross-coupling reaction in DES.

The effectiveness of Songashira reaction was evaluated using various DESs. Among them, the combination of Ph_3_PMeBr:glycerol (1:2) demonstrated the most favorable outcome when conducted at a temperature of 60 °C for a duration of 16 h, employing ^i^Pr_2_NH (2 equiv.) as the base. The reaction successfully generated the desired cross-coupled products **127** with moderate to high yields. This outcome was achieved by subjecting a range of aryl halides **125**, which incorporated electron-withdrawing groups, such as iodides and bromides, to the reaction conditions. However, aryl iodides containing electron-donating groups exhibited lower yields, suggesting the significant influence of the electronic properties of the aryl halides in this particular methodology. Notably, a moderate yield was also attained by cross-coupling the aliphatic terminal alkyne ethynylcyclohexane **126** with 1-iodo-4-nitrobenzene **125**. Furthermore, the introduction of an electron-rich thienyl derivative led to a favorable yield (Scheme 38).

DES catalytic systems were examined for recyclability using 2-MeTHF to extract organic compounds [125]. Five cycles were found to be effective in the Hiyama and Suzuki reactions, but only three cycles for Heck and Sonogashira reactions. This explains the catalyst’s decreasing efficiency, as confirmed by TEM and XPS analysis. In their discussion, the researchers stressed the importance of DES for stabilizing PdNPs, so that they remain active for multiple runs [126].

**Scheme 39:**
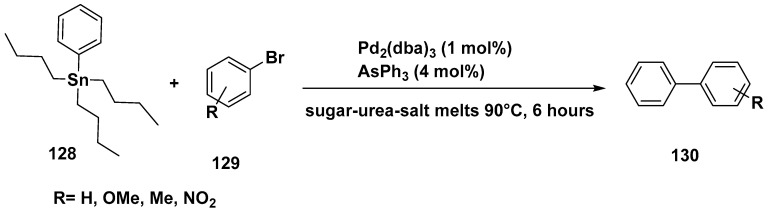
Stille Reactions with tetraalkylstannanes and phenyltrialkylstannanes in low melting mixture.

In a study by Imperato et al., Stille reactions were performed using tetraalkylstannanes and phenyltrialkylstannanes in a eutectic mixture of Sugar–Urea–Salt [127]. Various aryl bromides **129** reacted with tributylphenylstannane **128** in the presence of 1 mol% Pd_2_(dba)_3_ as the catalyst and 4 mol% AsPh_3_ as the ligand, for six hours. The best results for biaryl synthesis were obtained using mixtures such as lactose:dimethylurea:NH_4_Cl (6:3:1), maltose:dime-thylurea:NH_4_Cl (5:4:1), mannitol:dimethylurea:NH_4_Cl (5:4:1), and sorbitol:dimethylurea:NH_4_Cl (7:2:1). This methodology was highly tolerant to para-substituted aryl bromides with electron-donating or electron-withdrawing groups, affording excellent to quantitative yields. The reaction mixture was easily worked up by treating it with water to dissolve the eutectic mixture and precipitate the desired products **130**. Notably, the catalyst retained its activity for three consecutive runs with only a slight decrease in conversion (Scheme 39).

**Scheme 40:**
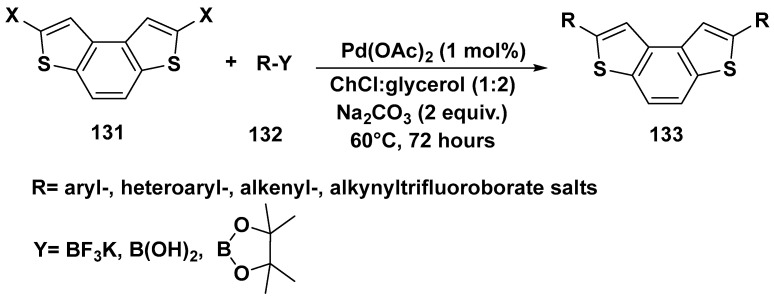
Suzuki coupling of benzodithiophene derivatives.

Pelliccioli and colleagues found that dihalogeno-substituted benzodithiophenes (BDTs) can undergo a Suzuki–Miyaura cross-coupling reaction without the need for ligands. This reaction occurs under moderate heating (60 °C) and in the presence of air [128]. The symmetric arylation and alkenylation of benzodithiophene dihalides **131** smoothly proceeded using Pd(OAc)_2_ (1 mol%) as a catalyst and Na_2_CO_3_ (2 equiv.) as a base in the mixture of ChCl:glycerol (1:2) for 72 h, yielding products **133** with potential electrochromic properties (Scheme 40). The alkynylation was also feasible using this strategy, affording the desired product **133** in moderate yield. The described conditions were recently reported by Capriati and co-workers, and ChCl:glycerol (1:2) proved to be compatible under air conditions in the absence of a ligand [71]. The optical and electrochemical properties of these systems have been thoroughly investigated by means of absorption and cyclic voltammetry measurements.

**Scheme 41:**
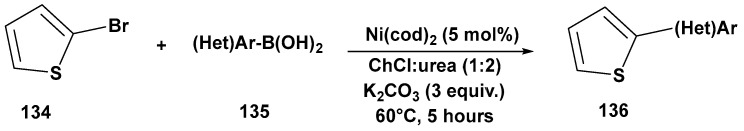
Ni-catalyzed Suzuki–Miyaura cross-coupling.

In 2020, a study reported on the use of a DES in the Suzuki–Miyaura coupling reaction using a Ni-catalyst instead of a Pd-catalyst [129]. The reaction was carried out with 5 mol% of Ni(cod)_2_ and involved the reaction of 2-bromothiophene **134** with various arylboronic acids **135** in a ChCl:urea (1:2) solvent mixture, with potassium carbonate as a base. The reaction was conducted for five hours at 60 °C, resulting in good-to-excellent yields for all 20 examples reported **136** (Scheme 41). The scaffolding of the synthesized products proved to have significant biological activity [130]. DESs can interact with boronic acids by forming hydrogen bonds with the acidic OH group of the boronic acid, promoting their deprotonation [131]. DESs can also facilitate the transmetalation step of the reaction by stabilizing the metal catalyst and promoting the formation of a boronate-bridged intermediate that is more reactive towards the aryl halide. The high polarity of DESs and their ability to coordinate with metal catalysts can help to stabilize the catalytic species, prevent catalyst deactivation, and promote the desired reaction pathway. The solvent and catalyst can be reused for several consecutive times without a significant loss in activity.

## 4. Conclusions

Over a decade ago, DESs were introduced as a potential alternative to traditional volatile organic solvents in organic synthesis. Initially, DESs faced limitations due to their differing physicochemical properties from traditional solvents. However, today they have become a viable option for synthetic methods. Certain DESs that contain a metal salt in their structure, can be used as recyclable solvents and catalysts for several C–H activation and cross-coupling transformations. Recently, improvements in knowledge about the compatibility of homogeneous and heterogeneous metallic catalysts in DESs have greatly enhanced their efficiency and selectivity. Consequently, DESs are no longer limited to specific reactions, but have proven to be useful alternatives to traditional solvents in transition-metal-catalyzed organic transformations. The recovery and recycling of the catalyst/DES system have significantly improved reaction efficiencies. DESs offer several advantages, such as increased sustainability, reduced energetic costs, and the ability to perform reactions at milder conditions. Additionally, DESs have shown unique properties that lead to novel and exciting reactivities. However, the proper selection of DES and the correct design of the metal-ligand system are essential for successful outcomes. DESs are gaining popularity, and the knowledge and applications of these solvents will continue to grow in the future.

## Data Availability

Not applicable.
